# Computational Study on New Natural Compound Inhibitors of Pyruvate Dehydrogenase Kinases

**DOI:** 10.3390/ijms17030340

**Published:** 2016-03-04

**Authors:** Xiaoli Zhou, Shanshan Yu, Jing Su, Liankun Sun

**Affiliations:** 1 College of Basic Medical Sciences, Jilin University, Changchun 130021, Jilin, China; zhouxl@jlu.edu.cn (X.Z.); sujing@jlu.edu.cn (J.S.); 2 State Key Laboratory of MicrobialMetabolism, School of Life Sciences and Biotechnology, Shanghai Jiao Tong University, Shanghai 200240, China; yushanshan001@aliyun.com; 3 Jilin Ginseng Academy, Changchun University of Chinese Medicine, Changchun 130000, Jilin, China

**Keywords:** pyruvate dehydrogenase kinase, inhibitor, virtual screening, coumarins

## Abstract

Pyruvate dehydrogenase kinases (PDKs) are key enzymes in glucose metabolism, negatively regulating pyruvate dehyrogenase complex (PDC) activity through phosphorylation. Inhibiting PDKs could upregulate PDC activity and drive cells into more aerobic metabolism. Therefore, PDKs are potential targets for metabolism related diseases, such as cancers and diabetes. In this study, a series of computer-aided virtual screening techniques were utilized to discover potential inhibitors of PDKs. Structure-based screening using Libdock was carried out following by ADME (adsorption, distribution, metabolism, excretion) and toxicity prediction. Molecular docking was used to analyze the binding mechanism between these compounds and PDKs. Molecular dynamic simulation was utilized to confirm the stability of potential compound binding. From the computational results, two novel natural coumarins compounds (ZINC12296427 and ZINC12389251) from the ZINC database were found binding to PDKs with favorable interaction energy and predicted to be non-toxic. Our study provide valuable information of PDK-coumarins binding mechanisms in PDK inhibitor-based drug discovery.

## 1. Introduction

Pyruvate dehydrogenase kinases (PDKs) are key enzymes in glucose metabolism since they negatively regulate the activity of pyruvate dehydrogenase complex (PDC) by phosphorylation [1,2]. PDC is an important gatekeeper enzyme that catalyzes the oxidative decarboxylation of pyruvate to produce acetyl CoA [3]. The mammalian PDC is a 9.5 million Dalton multi-enzyme complex consisting of pyruvate dehydrogenase (E1), dihydrolipoyltransacetylase (E2), dihydrolipoamide dehydrogenase (E3) and the E3-bindingprotein (E3BP). PDKs are non-covalently bound to the lipoyl domain (L2) of the E2 subunit of PDC. They phosphorylate the pyruvate dehydrogenase (PDH)-E1α and inactivate PDC [4,5,6,7]. In mammalians, there are four isoenzymes of PDKs (PDK 1 to 4) with different binding affinity, phosphorylation site specificity and tissue distribution [8,9]. The binding affinities of four isoenzymes to L2 are in the following order: PDK3 > PDK1 ≈ PDK2 > PDK4. As for the phosphorylation sites (S264, S271 and S203), only PDK1 is reported to phosphorylate all three sites, while other isoenzymes can only phosphorylate S264 and S271 with different rates. Four isoenzymes have different tissue distribution: PDK1 is present preferentially in the pancreatic islets, heart and skeletal muscles; PDK2 is expressed universally in all tissues; PDK3 is abundant in the kidney, brain and testes; PDK4 is more easily detected in the pancreatic islets, heart, kidney and skeletal muscle.

The PDKs are potential therapeutic targets since the up-regulation of PDKs are associated with many diseases such as cancer, diabetes, obesity and heart failure [10,11,12,13,14]. Several recent studies have provided evidence that PDKs play major roles in the metabolism of cancer cells, since the inactivation of PDC is tightly associated with the “Warburg effect” [15,16]. It has been reported that, PDKs are universally overexpressed in a variety of cancer cells, such as multiple myeloma and breast cancer. Moreover, the expression and activities of the PDKs are strongly regulated by oncogenes [17,18,19]. Type 2 diabetes and obesity are characterized by dysregulation of glucose and lipid metabolism, in which PDC plays a pivotal role [20]. Increasing the activity of PDC through inhibition of PDKs could improve glucose oxidation and reduce the blood glucose concentration, thus ultimately promoting insulin activity. Thus, development of potent PDK inhibitors could provide powerful treatment for cancers and metabolic diseases.

In recent years, small molecule inhibitors with different binding sites have been reported to regulate the activities of PDKs [21]. Dichloroacetate (DCA) is a structural analog of pyruvate, binding to the regulatory domain of PDKs to regulate their activities [22,23]. AZD7545 binds to the lipoamide-binding site of PDKs, and disrupts the interaction between PDKs and PDC component, increasing the activity of PDC indirectly [24,25]. Radicicol and M77976 bind to the nucleotide-binding pocket and inactivate PDKs by blocking ATP entry to the pocket [26]. There are also some other small molecule inhibitors with similar mechanisms, such as JX06 [27] and VER-246608 [28]. Among all these inhibitors, only DCA has entered clinical trials. Although significant progress has been made in the research of PDK inhibitors with biological activity, design and development of compounds with novel skeleton for therapeutic use are still focus of attention.

The use of natural products has a long history in medicine. Natural products and their derivatives represent a major part of today’s pharmaceutical market [29,30]. They also play an important role as tool compounds in molecular biological research, such as pathway screening and validation of target identification [31,32,33]. With the advantage of their innate affinity for biological receptors, natural compounds may provide a valuable source for the development of PDK-based drugs. To discover new PDKs inhibitors, we carried out a virtual screening against the Natural Products database (NP) in the ZINCdatabase [34]. ADME (absorption, distribution, metabolism, excretion) and toxicity properties of the obtained screened compounds were analyzed to examine drug like properties. Binding modes of the selected candidate compounds obtained from the docking were studied by visual inspection and their binding stabilities were further evaluated by performing 40 ns molecular dynamics simulation via Discovery Studio 3.5.

## 2. Results

### 2.1. Virtual Screening of Natural Products Database against Pyruvate Dehydrogenase Kinase 1 (PDK1)

Lipoamide-binding pocket is an important regulatory site of PDKs, since small molecules binding to this pocket can prevent PDKs from binding to PDC but with minimal effect on the function of the enzyme complex. To identify new compounds that could potentially inhibit PDK1 through binding to the lipoamide-binding pocket, a virtual screening was carried out using Libdock [35] module of Discovery Studio 3.5 (DS3.5, Accelrys, Inc., San Diego, CA, USA). 99,932 purchasable natural product molecules were taken from the ZINC database, which is a free database of commercially-available compounds provided by the Irwin and Shoichet Laboratories in the Department of Pharmaceutical Chemistry at the University of California, San Francisco (UCSF). The target used in this study was the 3D structure of PDK1 (PDB ID: 2Q8G) [25]. The well known PDK inhibitor AZD7545, which can inhibit PDKs activity except PDK4 *in vitro* and *in vivo* [24,36], was chosen as reference compound. AZD7545 inhibited PDK1 through the trifluoromethylpropanamide end that inserted into the lipoamide-binding pocket of PDK1, as revealed by the crystal structure of human PDK1-AZD7545 complex. The blocking of the lipoamide-binding pocket resulted in inhibition of PDKs activities by aborting kinase binding to the PDC scaffold [25,37,38]. After the screening, 2354 compounds were found have higher Libdock scores than AZD7545 (Libdock score: 117.276). The top 20 ranked compounds are listed in Table 1.

### 2.2. ADME (Adsorption, Distribution, Metabolism and Excretion) Properties and Toxicity Prediction

ADME for all the selected ligands and AZD7545 were predicted using the ADMET module of DS, including brain/blood barrier (BBB), human intestinal absorption, aqueous solubility, cytochrome P450 2D6 (CYP2D6) binding, hepatotoxicity and plasma protein binding properties (PPB) (Table 2). The aqueous solubility prediction (defined in water at 25 °C) indicated that all the compounds are soluble in water. For human intestinal absorption, 11 compounds and AZD7545 had a good absorption level, and four compounds had a moderate absorption level. All compounds were found to be highly bound with plasma protein except ZINC08878685. Thirteen compounds were predicted to be non-inhibitors of cytochrome P450 2D6 (CYP2D6), which is one of the important enzymes involved in drug metabolism. For hepatotoxicity, seven compounds were predicted non-toxic in comparison to AZD7545 (toxic).

**Figure 1:**
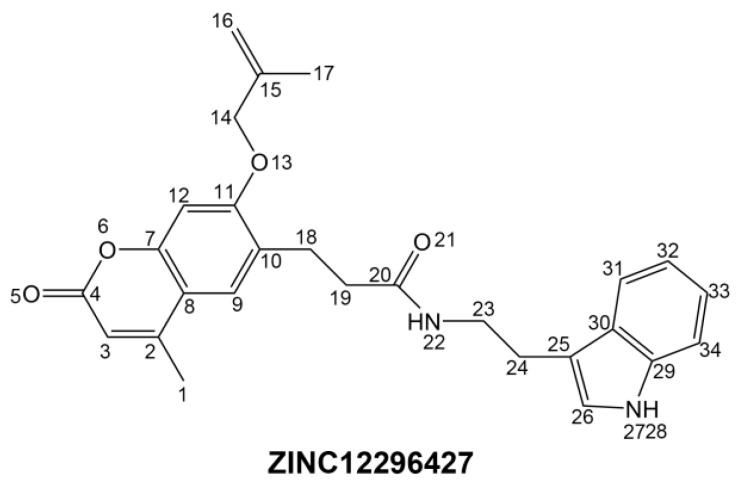

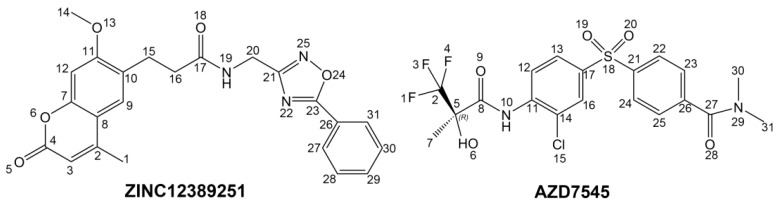
The structures of the novel compounds from virtual screening and the reference AZD7545.

Safety is an important aspect of drug research. To examine the safety of the compounds, different toxicity such as Ames mutagenicity (AMES), rodent carcinogenicity (based on the U.S. National Toxicology Program (NTP) dataset) and developmental toxicity potential (DTP) properties of compounds and AZD7545 were predicted using TOPKAT module of DS (Table 3). The results showed that, all compounds were predicted to be non-mutagen. Eleven compounds were predicted to be non-carcinogen and seven compounds with no developmental toxicity potential. The reference AZD7545 was predicted with developmental toxicity potential. Synthesizing the above results, ZINC12296427 and ZINC12389251 are not CYP2D6 inhibitors, with no hepatotoxicity. Moreover, they are predicted with no Ames mutagenicity, rodent carcinogenicity and developmental toxicity potential. Therefore, ZINC12296427 and ZINC12389251 were predicted safe drug candidates and selected for further research (Figure 1).

### 2.3. Ligand Binding Analysis

**Figure 2:**
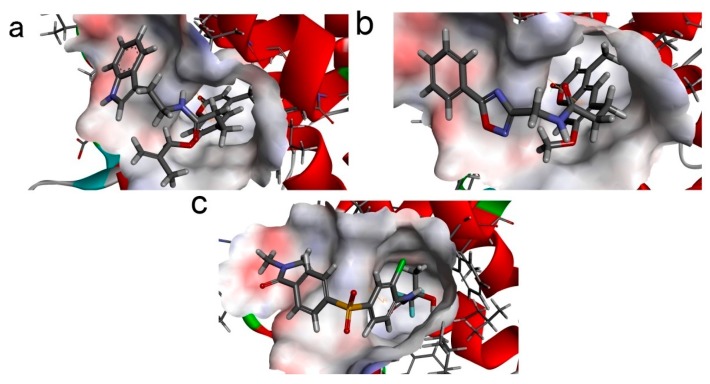
Schematic drawing of interactions between inhibitors and PDK1. The surface of lipoamide-binding pockets are shown, blue represents positive charge, red represents negative charge. Inhibitors are shown in sticks. (**a**) ZINC12296427-PDK1 complex; (**b**) ZINC12389251-PDK1 complex; (**c**) AZD7545-PDK1 complex.

**Figure 3:**
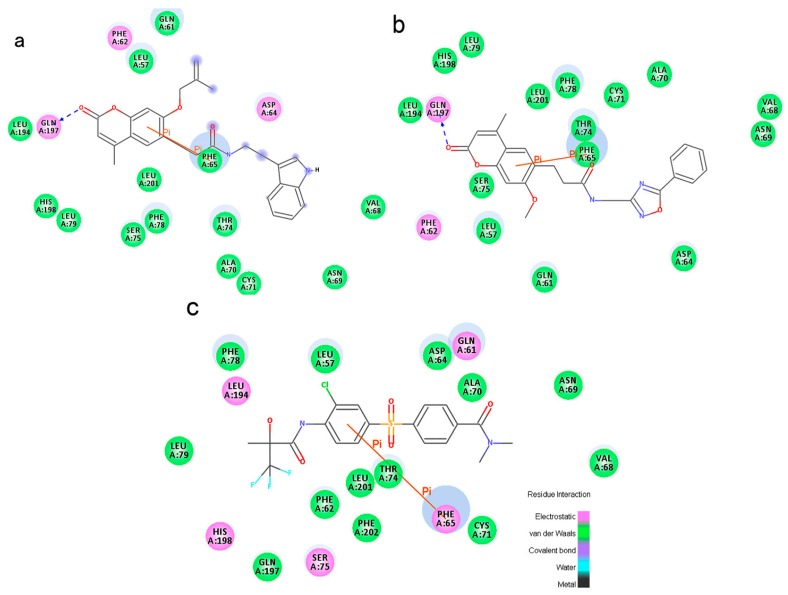
The inter-molecular interaction of the predicted binding modes of (**a**) ZINC12296427; (**b**) ZINC12389251 and (**c**) AZD7545 to PDK1.

In order to study ligand binding mechanisms, ZINC12296427 and ZINC12389251 were docked into the 3D structure of PDK1 by using the CDOCKER module of DS [39]. The CDOCKER interaction energy as an estimation of molecular complex binding affinity was used in this study. As shown in Table 4, the CDOCKER interaction energy of ZINC12296427 is lower than the reference AZD7545, indicating that ZINC12296427 may have a higher binding affinity with PDK1. The energy of the other compound ZINC12389251 is close to AZD7545. We analyzed the hydrogen bonds and Pi-Pi interactions formed by PDK1 and these compounds (Figure 2 and Figure 3, Table 5 and Table 6). The results showed that, both ZINC12296427 and ZINC12389251 formed one pair of hydrogen bond with PDK1, by the carbonyl O of compounds and the amide -NH of GLN197 of PDK1. No hydrogen bonds were formed between AZD7545 and PDK1. Each of the three compounds formed one pair Pi-Pi interaction with PDK1, by the centroids of the benzene ring of PHE65 and the centroids of the benzene ring of coumarin groups of ZINC12296427 and ZINC12389251 or chlorinated benzene ring of AZD7545, respectively. The additional hydrogen bond formed between ZINC12296427, ZINC12389251 and the lipoamide-binding pocket of PDK1might contribute to the lower interaction energy of the complexes. These results indicated that, ZINC12296427 and ZINC12389251 probably bind to PDK1 with similar or even better affinity than AZD7545.

**Figure 4:**
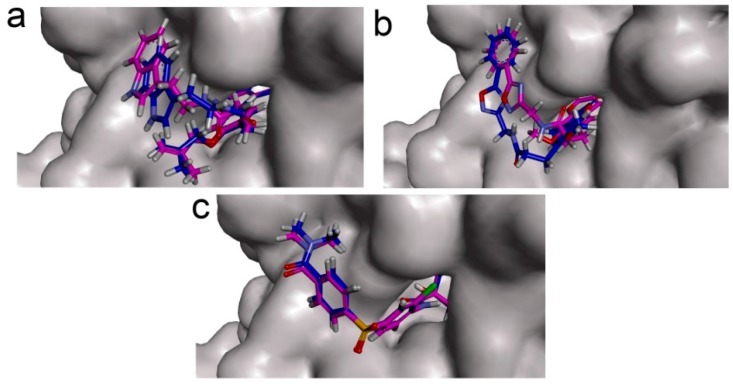
Superimposed structures of PDK1-docked compounds using CDOCKER (purple) and AutoDock (blue). (**a**) ZINC12296427; (**b**) ZINC12389251; (**c**) AZD7545. The surface of lipoamide binding pocket of PDK1 are shown in grey.

To confirm the docking analysis performed with CDOCKER, the docking results were crosschecked using AutoDock [40]. All docking conformations were visualized using DS3.5 so as to ensure the ligands were docked into the defined binding pocket. The superimposed docked structures are shown in Figure 4. For ZINC12296427 and AZD7545, the docked poses generated by AutoDock were similar to the poses generated by CDOCKER, with RMSD (Root Mean Square Deviation) values of 1.5 and 0.3 Å, respectively. ZINC12296427 had a lower binding energy (−8.8 kcal/mol) than AZD7545 (−6.3 kcal/mol) (Table 7), in accordance with the results of CDOCKER. There were some differences between the docked poses of ZINC12389251 generated by AutoDock and CDOCKER. In the PDK1-ZINC12389251 complex predicted by AutoDock, there was one more pair of Pi-Pi interaction compared to the results of CDOCKER, formed by the centroids of the benzene ring of PHE65 and the coumarin group of ZINC12389251.This extra pair of Pi-Pi interaction might contribute to the affinity of ZINC12389251 with PDK1, making ZINC12389251 have the lowest binding energy (−9.3 kcal/mol) in three compounds (Table 7). Overall, both ZINC12296427 and ZINC12389251 bind to lipoamide binding pocket of PDK1 with lower energy than AZD7545 predicted by the two methods.

### 2.4. Molecular Dynamics Simulation

**Figure 5:**
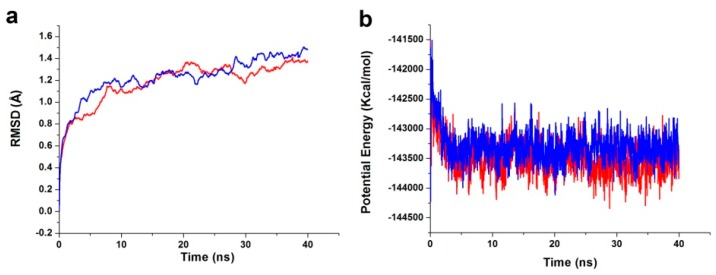
Results of MD simulation of two complexes. (**a**) Average backbone RMSD; (**b**) Potential Energy. Red, ZINC12296427-PDK1; blue, ZINC12389251-PDK1.

To evaluate the stabilities of PDK1-ligand complexes under dynamic conditions, we conducted the molecular dynamics (MD) simulation using DS. The initial conformations were acquired from the molecular docking experiments by CDOCKER. The RMSD curves of the receptor structures from each complex and the potential energy profiles of each complex are shown in Figure 5. The trajectories of complexes reached equilibrium after 10 ns, RMSD and potential energy of the complexes gets stabilized with the time. The H-bond distances formed between ZINC12296427, ZINC12389251 and PDK1 were within a range around 3.0 and 2.3 Å. In addition, both ZINC12296427 and ZINC12389251 formed two pairs of hydrogen bonds with the water molecules after molecular dynamics simulation (TIP21914:H2-ZINC12296427:O21; ZINC12296427:H28-TIP13772:OH2; TIP12999:H2-ZINC12389251:O18; ZINC12389251:H45-TIP17204:OH2). These hydrogen bonds might contribute to the stability of the complexes. Taking all the evaluation indexes into consideration, these two compounds might interact with PDK1 steadily and have potential negative modulatory effects on PDK1.

### 2.5. Molecular Docking with Other PDKs

ZINC12296427 and ZINC12389251 were docked into the 3D structures of PDK2, PDK3 and PDK4 to evaluate if they could inhibit other PDKs. CDOCKER and AutoDock results showed that, except for PDK4, both compounds could bind to the lipoamide binding pockets of PDK2 and PDK3 with strong interaction energy (Table 4 and Table 7), and formed hydrogen bond (Table 5) and Pi-Pi interactions (Table 6) with receptors. These results indicated that ZINC12296427 and ZINC12389251 might be broad spectrum inhibitors of PDKs.

## 3. Discussion

To phosphorylate the pyruvate dehydrogenase (E1 of PDC), PDKs need to interact with the L2 domain of dihydrolipoyltransacetylase (E2 of PDC) through their lipoamide binding pockets. Inhibitors binding to the lipoamide binding pockets can uncouple the link between PDKs and PDC, and then upregulate PDC activity indirectly. In this study, we identified two novel natural compounds, ZINC12296427 and ZINC12389251, from ZINC database to effectively inhibit PDKs through virtual screening technique. Molecular docking study showed that the two novel compounds could bind to the lipoamide-binding pocket of PDKs through forming of hydrogen bonds and Pi-Pi interactions with receptors. Moreover, these two compounds were predicted with no hepatotoxicity, Ames mutagenicity, rodent carcinogenicity and developmental toxicity. Thus, our findings provided potential mechanism of two novel inhibitors of PDKs for clinical drug design. Since the compounds were virtually screened and their inhibitory activities have not been reported, experiments such as IC_50_ and EC_50_ measurements will be carried out in our further study to examine their bioactivities.

Our results of the binding mechanism study also showed that the functional groups of these two compounds are their coumarins group. Coincidentally, recent studies have reported the antitumor activities of coumarins and their derivatives [41]. Our results suggest that upregulation of mitochondria aerobic metabolism of cancer cells through inhibition of PDKs might contribute to the anticancer activities of this kind of compound. This is also probably one possible mechanism for the anticancer activity of coumarones, which have similar structure to coumarins. Further studies are needed to determine the exact mechanism. Coumarins have broad pharmacological activities, such as anti-coagulant, anti-viral, anti-inflammatory and anti-microbial effects [42]. Therefore, development of novel bioactivities of natural and synthetic coumarins based on their PDKs inhibition activities is of great significance for the treatment of metabolic diseases.

## 4. Materials and Methods

### 4.1. Docking Software and Ligand Library

Libdock, and ADMET modules of Discovery Studio 3.5 software (DS3.5, Accelrys, Inc.) were used for virtual screening and ADMET analyzing of inhibitors. CDOCKER and AutoDock were used for docking study. A Natural Products database (NP) in the ZINC database was employed to screen PDKs inhibitors.

### 4.2. Structure-Based Virtual Screening Using Libdock

Libdock is a rigid-based docking program. It calculates hotspots for the protein using a grid placed into the binding site and using polar and apolar probes (San Diego, CA, USA). Then the hot spots are further used to align the ligands to form favourable interaction. The Smart Minimiser algorithm and CHARMm force field (Cambridge, MA, USA) were used for ligands minimization. After minimized, all the ligand poses are ranked based on the ligands score. The 1.9 Å crystal structure of human pyruvate dehydrogenase kinase 1 (PDK1) in complex with AZD7545 (PDB ID: 2Q8G) were downloaded from protein data bank (PDB) and imported to the working environment of Libdock. The protein was prepared by removing crystal water and other hetero atoms (except AZD7545), followed by addition of hydrogen, protonation, ionization and energy minimization. The CHARMm [43] force field and the Smart Minimiser algorithm were applied for energy minimization. The minimization performed 2000 steps with an RMS gradient tolerance of 0.1, and the final RMS gradient was 0.09463. The prepared protein was used to define the binding site from the “Edit binding site” option on the receptor-ligand interaction tool bar. Using the bound ligands (AZD7545) binding positions, the active sites for docking were generated. Virtual screening was carried out by docking all the prepared ligands at the defined active site using Libdock. Based on the Libdock score, all the docked poses were ranked and grouped by name. All compounds were ranked according to their Libdock score.

### 4.3. Molecular Docking

CDOCKER module of Discovery Studio was used for molecular docking study. CDOCKER is an implementation of a CHARMm based docking tool. The receptor is held rigid while the ligands are allowed to flex during the docking process. For each complex pose, the CHARMm energy (interaction energy plus ligand strain) and the interaction energy, which indicate ligand binding affinity, are calculated. Crystal structure of PDK1 (PDB ID: 2Q8G, 1.9 Å), PDK2 (PDB ID: 2BU6, 2.4 Å) [44], PDK3 (PDB ID: 2Q8I, 2.6 Å) [25] and PDK4 (PDB ID: 2ZDX, 2.5 Å) [26] were obtained from the protein data bank. The crystal water molecules are generally removed in rigid and semi-flexible docking process [45,46], since the fixed water molecules might affect the formation of receptor-ligand complex. The water molecules were removed and hydrogen atoms were added to the protein. The 3D structures of ZINC12296427 and ZINC12389251 were obtained from ZINC database (no experimental crystal structures available in crystallographic databases). The CHARMm forcefield was used for receptors and ligands. The binding site spheres of PDK1 and PDK2 were defined as the regions that come within radius 15 Å from the geometric centroid of the ligands AZD7545, AZ12, respectively. The binding site spheres of PDK3 and PDK4 were defined by structural alignment with PDK1. During the docking process, the ligands were allowed to bind to the residues within the binding site spheres. After being extracted from the binding site, the initial compound AZD7545 was re-docked into the crystal structure of PDK1. The RMSD between the docked pose and the crystal structure of the complex was 0.6 Å, indicating the CDOCKER module was highly reliable for reproducing the experimentally observed binding mode of PDKs. The structures of identified hits were prepared and docked into the lipoamide-binding pocket of PDKs. Different poses for each test molecule were generated and analyzed on the basis of CDOCKER interaction energy.

AutoDock 4.2 (The Scripps Research Institute, (La Jolla, CA, USA) was used to crosscheck the docking results of CDOCKER. The AutoDockTools 1.5.6 was used to prepare the protein and ligands for docking procedure. All solvent molecules, water molecules, and the cocrystallized ligand were removed from the structure; Kollman charges and polar hydrogens were added. AutoGrid (La Jolla, CA, USA) was used to generate the grid maps. Each grid was centered at the lipoamid binding site of the PDKs. The grid dimensions were 45 points in each dimension separated by 0.375 Å. The files were generated as PDBQT format. For all ligands, random starting positions, orientations and torsions were used. PDBQT file of the ligands was generated with all the default values accepted. The Genetic Algorithm was used with 2,500,000 energy evaluations and a population of 300 individuals; 100 runs were carried out. Following docking, the results were clustered into groups with RMSD <2.0 Å. The ranking of the clusters was performed based on the lowest energy representative of each cluster. Visualization and analysis of interactions between protein and ligand were performed using DS3.5.

### 4.4. Molecular Dynamics Simulation

The best binding conformations of the PDK1-inhibitor complexes among the poses predicted by the molecular docking program were selected and used as MD simulation starting points. The ligand-receptor complex was put into an orthorhombic box and solvated with an Explicit Periodic Boundary solvation water model. The size of the box was 88 × 75 × 72 Å, containing 13,802 water molecules. In order to simulate the physiological environment, sodium chloride were added to the system with the ionic strength of 0.145. Then, the system was subjected to the CHARMm force-field and relaxed by energy minimization (1000 steps of steepest descent and 1000 steps of conjugated gradient), with the final RMS gradient of 0.08326. The system was slowly driven from an initial temperature of 50 K to the target temperature of 300 K for 2 ns and equilibration simulations were run for 1 ns. The MD simulations (production) were performed for 40 ns with time step of 2 fs. The simulation was performed with the NPT (normal pressure and temperature) system at a constant temperature of 300 K and the results were saved at a frequency of 0.02 ns. The Particle Mesh Ewald (PME) algorithm was used to calculate long range electrostatics, and the linear constraint solver (LINCS) algorithm was adapted to fix all bonds involving hydrogen. The initial complex was set as a reference. The MD trajectory was determined for structural properties, root mean-square deviation (RMSD), and potential energy by using the Discovery Studio 3.5 analyze trajectory protocol (San Diego, CA, USA).

### 4.5. ADME and Toxicity Prediction

The ADMET and TOPKAT modules of DS were employed to calculate the ADMET properties of the compounds, such as their aqueous solubility, blood-brain barrier (BBB) penetration, cytochrome P450 2D6 (CYP2D6) inhibition, hepatotoxicity, human intestinal absorption, plasma protein binding (PPB) level, rodent carcinogenicity, Ames mutagenicity and developmental toxicity potential.

## 5. Conclusions

The present study elucidates the possible inhibitory activity of two natural compounds ZINC12296427 and ZINC12389251 target PDKs. The ADMET properties of these compounds were predicted to be non-carcinogenic and non-toxic, indicating safe drug candidates. The results of this study not only demonstrate the probable binding mode of these compounds with PDKs, but also encourage further investigations of the effect of coumarins on metabolism through inhibition of PDKs.

## Figures and Tables

**Table 1:** Top 20 ranked compounds with higher Libdock scores than AZD7545.

Number	Compounds	Libdock Score	Number	Compounds	Libdock Score
1	ZINC12296427	156.581	11	ZINC11869091	149.303
2	ZINC12296380	155.357	12	ZINC12388848	149.092
3	ZINC12296441	154.649	13	ZINC12322380	148.82
4	ZINC12296745	153.371	14	ZINC12389251	148.308
5	ZINC70680971	152.663	15	ZINC12297108	147.747
6	ZINC12296825	151.658	16	ZINC20478854	147.695
7	ZINC12296957	151.201	17	ZINC12321394	147.052
8	ZINC11868961	151.117	18	ZINC11868932	147.019
9	ZINC70686684	149.856	19	ZINC20677981	146.944
10	ZINC22854522	149.306	20	ZINC08878685	146.804

**Table 2:** ADMET (Adsorption, Distribution, Metabolism and Excretion) properties of compounds.

Number	Compounds	Solubility Level ^a^	Absorption Level ^b^	BBB Level ^c^	Hepatotoxity ^d^	CYP2D6 ^e^	PPB Level ^f^
1	ZINC12296427	1	1	4	0	0	2
2	ZINC12296380	1	0	4	0	1	2
3	ZINC12296441	1	1	4	1	0	2
4	ZINC12296745	2	0	2	1	0	2
5	ZINC70680971	1	2	4	1	0	2
6	ZINC12296825	2	1	4	0	0	2
7	ZINC12296957	1	1	4	1	1	2
8	ZINC11868961	1	2	4	1	1	2
9	ZINC70686684	1	2	4	1	0	2
10	ZINC22854522	3	0	1	1	1	1
11	ZINC11869091	1	2	4	1	0	2
12	ZINC12388848	2	0	2	1	0	2
13	ZINC12322380	2	0	4	1	0	2
14	ZINC12389251	2	0	3	0	0	2
15	ZINC12297108	2	0	2	0	1	2
16	ZINC20478854	2	0	2	1	1	2
17	ZINC12321394	2	0	1	0	0	2
18	ZINC11868932	1	2	4	1	0	2
19	ZINC20677981	2	0	1	0	0	2
20	ZINC08878685	3	0	3	1	1	0
21	AZD7545	2	0	4	1	0	1

^a^ Aqueoussolubility level: 0 (extremely low); 1 (very low, but possible); 2 (low); 3 (good); ^b^ Humanintestinal absorption level: 0 (good); 1 (moderate); 2 (poor); 3 (very poor); ^c^ Blood Brain Barrier level: 0 (Very high penetrant); 1 (High); 2 (Medium); 3 (Low); 4 (Undefined); ^d^ Hepatotoxicity: 0 (Nontoxic); 1 (Toxic); ^e^ Cytochrome P450 2D6 level: 0 (Non-inhibitor); 1 (Inhibitor); ^f^ Plasma Protein Binding: 0 (Binding is <90%); 1 (Binding is >90%); 2 (Binding is >95%).

**Table 3:** Toxicities of compounds.

Number	Compounds	Mouse NTP ^a^		Rat NTP ^a^		AMES ^b^	DTP ^c^
		Famale	Male	Famale	Male		
1	ZINC12296427	0	0	0	0	0	0
2	ZINC12296380	0	0	0	0	0	0
3	ZINC12296441	0	0	0	0	0	1
4	ZINC12296745	0	0	0	0	0	1
5	ZINC70680971	0	1	1	1	0	1
6	ZINC12296825	0	0	0	0	0	1
7	ZINC12296957	0	0	0	0	0	0
8	ZINC11868961	0	1	1	0	0	1
9	ZINC70686684	0	1	1	0	0	1
10	ZINC22854522	0	1	1	0	0	1
11	ZINC11869091	0	1	1	0	0	1
12	ZINC12388848	0	0	0	0	0	1
13	ZINC12322380	0	0	0	0	0	0
14	ZINC12389251	0	0	0	0	0	0
15	ZINC12297108	0	0	0	0	0	0
16	ZINC20478854	0	1	0	0	0	0
17	ZINC12321394	0	0	0	0	0	1
18	ZINC11868932	0	1	1	0	0	1
19	ZINC20677981	0	1	0	0	0	1
20	ZINC08878685	1	0	0	1	0	1
21	AZD7545	0	0	0	0	0	1

^a^ 0 (Non-Carcinogen); 1 (Carcinogen); ^b^ 0 (Non-Mutagen); 1 (Mutagen); ^c^ 0 (Non-Toxic); 1 (Toxic).

**Table 4:** CDOCKER interaction energy of compounds with pyruvate dehydrogenase kinases (PDKs).

Compounds	CDOCKER Interaction Energy (kcal/mol)		
	PDK1	PDK2	PDK3
ZINC12296427	−47.8215	−43.6484	−44.1419
ZINC12389251	−46.0384	−42.8	−42.4346
AZD7545	−46.2328	−43.0563	−43.0877

**Table 5:** Hydrogen bond interaction parameters for each compound and PDK residues.

Receptor	Compound	Donor Atom	Receptor Atom	Distances (Å)
PDK1	ZINC12296427	GLN197:HE21	ZINC12296427:O5	2.22
	ZINC12389251	GLN197:HE21	ZINC12389251:O5	2.30
PDK2	ZINC12296427	GLN163:HE21	ZINC12296427:O5	2.12
	ZINC12389251	GLN163:HE21	ZINC12389251:O5	2.15
	AZD7545	AZD7545:H32	SER41:OG	2.04
PDK3	ZINC12296427	LYS30:HZ1	ZINC12296427:O5	1.88
	ZINC12389251	LYS30:HZ1	ZINC12389251:O5	1.82
	AZD7545	AZD7545:H32	ASP34:OD2	2.24

**Table 6:** Pi-Pi interaction parameters for each compound and PDK residues.

Receptor	Compound	End 1	End 2	Distances (Å)
PDK1	ZINC12296427	PHE65	ZINC12296427	4.08
	ZINC12389251	PHE65	ZINC12389251	4.25
	AZD7545	PHE65	AZD7545	4.64
PDK2	ZINC12296427	PHE31	ZINC12296427	3.99
		PHE31	ZINC12296427	4.09
		PHE44	ZINC12296427	5.79
	ZINC12389251	PHE31	ZINC12389251	4.01
		PHE31	ZINC12389251	3.99
	AZD7545	PHE31	AZD7545	4.83
PDK3	ZINC12296427	PHE35	ZINC12296427	4.27
		PHE35	ZINC12296427	4.36
	AZD7545	PHE35	AZD7545	4.36
		PHE48	AZD7545	4.96

**Table 7:** Binding energy of compounds with PDKs predicted by AutoDock.

Receptors	Compounds	Binding Energy (kcal/mol)
PDK1	ZINC12296427	−8.8
	ZINC12389251	−9.3
	AZD7545	−6.5
PDK2	ZINC12296427	−8.4
	ZINC12389251	−7.5
	AZD7545	−6.9
PDK3	ZINC12296427	−9.1
	ZINC12389251	−7.8
	AZD7545	−6.8
